# Neural and Behavioral Evidence for Differential Processing of Narrative Perspective in Novel Reading: An fNIRS Study

**DOI:** 10.3390/bs16020190

**Published:** 2026-01-29

**Authors:** Lijuan Chen, Xiaodong Xu

**Affiliations:** 1 School of Foreign Studies, Nanjing University of Posts and Telecommunications, Nanjing 210023, China; 2 School of Foreign Languages and Cultures, Nanjing Normal University, Nanjing 210097, China

**Keywords:** narrative perspective, focalization mode, emotional valence, self-paced reading, functional near-infrared spectroscopy (fNIRS), novel comprehension

## Abstract

Narrative perspective and focalization mode constitute fundamental elements shaping readers’ cognitive and neural responses during novel comprehension. Despite their theoretical importance in narratology, empirical evidence for their distinct processing mechanisms remains limited. This study employed a multi-method approach combining self-paced reading (N = 103) and functional near-infrared spectroscopy (fNIRS; N = 37) to investigate how narrative perspective (first-person vs. third-person) and focalization mode (internal vs. external) influence reading processes, with emotional valence as a potential moderator. Behavioral results revealed significantly prolonged reading times for third-person narratives compared to first-person narratives, particularly in negatively valenced texts. This effect was most pronounced among individuals with higher social cognitive abilities (low Autism Spectrum Quotient scores). Neuroimaging findings demonstrated distinct neural signatures: first-person narration elicited enhanced activation in the left superior parietal lobule compared to third-person narration, suggesting heightened attentional engagement. Internal focalization triggered greater activation in the left frontopolar cortex relative to external focalization, with negatively valenced texts showing similar enhanced activation patterns in this region. These converging lines of evidence support theoretical distinctions between narrative perspectives and demonstrate that first-person narration possesses higher cognitive salience during processing, while internal focalization more effectively engages readers’ metacognitive and empathetic neural systems. The findings provide empirical validation for longstanding narratological debates and illuminate the neurocognitive architecture underlying literary comprehension.

## 1. Introduction

Novel reading represents a unique form of cognitive and emotional engagement that extends beyond basic text comprehension to encompass complex processes of mental simulation, perspective-taking, and empathetic resonance. The narrative strategies employed in novels—particularly the manipulation of narrative perspective and focalization mode—fundamentally shape how readers construct mental representations of fictional worlds and connect with characters’ experiences. While narratological theory has long recognized these elements as central to literary meaning-making, empirical investigation of their cognitive and neural underpinnings has only recently emerged as a focus of interdisciplinary research bridging literature, psychology, and neuroscience. The present study addresses this gap by employing converging behavioral and neuroimaging methodologies to elucidate how different narrative perspectives and focalization modes modulate reading processes at both cognitive and neural levels, with particular attention to the role of emotional valence in shaping these effects. This investigation holds significant implications for understanding the mechanisms through which literary texts engage readers’ cognitive and affective systems, potentially informing both narratological theory and educational approaches to literary comprehension.

The distinctive characteristics of novel reading emerge from its demands on multiple cognitive systems. Unlike expository or informational texts, novels require readers to simultaneously process linguistic information, construct coherent situation models, track multiple character perspectives, and generate emotional responses to fictional events (Jacobs, 2015; Kidd & Castano, 2013; Mar et al., 2006; Tamir et al., 2016; Zunshine, 2006). This multifaceted engagement activates a distributed neural network encompassing traditional language processing regions alongside areas supporting theory of mind, episodic memory construction, and emotional regulation (Ferstl et al., 2008; Fletcher et al., 1995; Mar, 2011; Mason & Just, 2004; Speer et al., 2009).

Neuroimaging research has progressively mapped the neural architecture supporting narrative comprehension. Ferstl et al. (2008) demonstrated that discourse comprehension activates the prefrontal cortex, posterior cingulate cortex, and temporal lobes in patterns associated with situation model construction. Speer et al. (2009) revealed that different narrative events—including character actions, goals, and emotional transitions—selectively activate corresponding neural systems, including motor cortex for action simulation, and medial prefrontal cortex for mentalizing. Mar’s (2011) comprehensive review synthesized evidence showing that narrative comprehension extends beyond classical language areas (e.g., Broca’s and Wernicke’s regions) to engage the Theory of Mind network, highlighting the integration of language processing with memory, social cognition, and imagination in literary reading.

Despite these advances in understanding general narrative processing, the specific mechanisms through which narrative perspective and focalization mode influence reading remain underexplored. Novels employ sophisticated narrative strategies to guide readers’ engagement with story worlds, with perspective and focalization serving as primary tools for organizing narrative information and shaping reader-character relationships (Arfé et al., 2023). Narrative perspective, distinguished by grammatical person (first-person “I” versus third-person “he/she”), determines the vantage point from which events are narrated. Focalization mode, following Genette’s (1980) influential framework, specifies whether narrative information is filtered through a character’s consciousness (internal focalization) or presented from an external observer’s viewpoint (external focalization). These distinctions fundamentally alter the psychological distance between readers and fictional events, yet systematic experimental validation of their cognitive and neural effects remains limited.

### 1.1. The Influence of Narrative Perspective on Novel Reading

Narrative perspective represents a fundamental organizing principle in novel construction, with profound implications for reader engagement. First-person narration, employing the pronoun “I,” positions readers within the narrator’s subjective experience, establishing reading as a self-referential process. Third-person narration, utilizing “he/she” pronouns, constructs a more objective observational stance, positioning readers as external witnesses to narrative events (H. Chen, 2008). This distinction has generated extensive theoretical debate within narratology, crystallizing around the question of whether first-person and third-person narration represent fundamentally different modes of storytelling or merely surface variations in a single underlying structure.

The theoretical landscape reveals two opposing positions. Stanzel (1986) proposed a tripartite classification distinguishing omniscient, third-person, and first-person perspectives as distinct narrative modes with unique cognitive and rhetorical properties. This taxonomic approach, widely adopted in narratological scholarship, assumes substantive differences in how perspectives shape narrative meaning. Supporting this view, Forstreuter (1924) and Shen (1996) argue that first-person and third-person narration differ fundamentally in their focalization possibilities and rhetorical effects, rendering them non-interchangeable in most literary contexts. Conversely, Booth (1983) contends that surface grammatical distinctions mask underlying structural similarities, suggesting that perspective differences may be less significant than commonly assumed.

Empirical investigation of these theoretical positions has yielded mixed findings. The concept of cognitive salience—the degree to which information captures attention, undergoes deeper processing, and achieves better retention (Fiske & Taylor, 1991; Taylor & Fiske, 1978)—provides a framework for understanding perspective effects. Self-referential processing typically demonstrates enhanced speed, depth, and memory consolidation (Northoff et al., 2006), suggesting that first-person narration might possess inherent cognitive advantages through its activation of self-relevant processing mechanisms.

Recent neuroscientific evidence partially supports this hypothesis. At the sentence level, Magnetoencephalography (MEG) and transcranial magnetic stimulation (TMS) studies demonstrate that first-person sentences elicit stronger motor-related potentials and neural oscillatory responses compared to third-person equivalents (Niccolai et al., 2021; Papeo et al., 2011), indicating enhanced attention capture and interest generation. L. Chen et al. (2023, 2025) and Ditman et al. (2010) found that first-person narration reduces psychological distance and enhances emotional resonance. Hartung et al. (2016) demonstrated that pronoun manipulation significantly affects readers’ experiential engagement with narrative content.

However, other findings present a more complex picture. While Brilmayer et al. (2019) reported larger P300 components for first-person narratives—suggesting enhanced cognitive salience and attentional allocation—subsequent studies have reported mixed or null effects. Dixon et al. (2020) found no consistent advantage for first-person narration across multiple measures. Most notably, Hartung et al. (2017) failed to detect significant differences in neural activation patterns between perspectives across frontal, temporal, and occipital regions, raising questions about whether perspective differences manifest consistently at the neural level. These discrepancies likely reflect several methodological factors, including variations in stimulus materials (e.g., brief constructed texts versus extended literary excerpts), experimental paradigms (e.g., sentence-level versus discourse-level processing), dependent measures (e.g., reading times versus neural activation), and the inherent complexity of controlling for confounding variables in naturalistic literary texts. This underscores the need for systematic investigation using converging methodologies with carefully selected literary materials.

### 1.2. Additional Factors Influencing Novel Reading: Focalization Mode and Emotional Valence

Beyond narrative perspective, focalization mode represents another critical dimension shaping readers’ access to story information. Following Genette’s (1980) influential taxonomy, internal focalization restricts narrative information to a specific character’s knowledge, perceptions, and thoughts, positioning that character as the “focalizer” through whom events are filtered. This mode emphasizes subjective experience, providing direct access to characters’ mental and emotional states. External focalization, conversely, limits narration to externally observable behaviors and environmental descriptions, with the narrator serving as an objective focalizer who reports events without accessing characters’ consciousness (L. Chen et al., 2023). This distinction fundamentally alters the depth and nature of psychological information available to readers.

Despite its theoretical importance, empirical research on focalization mode remains surprisingly sparse. Wimmer et al. (2021) investigated focalization effects on social and moral cognition using post-reading questionnaires but found no significant differences between internal and external focalization conditions. This null finding may reflect limitations of offline measurement approaches, which cannot capture real-time processing dynamics. The interaction between focalization mode and narrative perspective remains particularly underexplored, despite theoretical predictions that these dimensions should interact to shape reading experiences.

Emotional valence constitutes an additional factor modulating narrative processing. Negative emotions demonstrate higher arousal potential than positive or neutral emotions, enhancing attention, analytical processing, and memory consolidation (L. Chen & Xu, 2020; Mouw et al., 2019). Burton et al. (2004) documented superior recall for negatively valenced texts compared to neutral alternatives, suggesting that negative emotional content facilitates comprehension and retention. This processing advantage may amplify differences between narrative perspectives under emotionally charged conditions.

Child et al. (2020) provided direct evidence for emotional moderation of perspective effects. In their eye-tracking study, negatively valenced texts produced distinct processing patterns: third-person pronouns initially required longer processing times than second-person pronouns, but this pattern reversed as reading progressed, with third-person processing becoming increasingly efficient. No such dynamic emerged for positively valenced texts, suggesting that negative emotional contexts specifically amplify perspective-based processing differences. This finding aligns with theoretical predictions that emotional intensity enhances sensitivity to narrative structural features.

In summary, while narrative perspective and focalization mode shape the novel reading process and produce distinct reading experiences, current research remains primarily at the level of textual analysis and theoretical exploration, with relatively limited systematic experimental investigation (e.g., Hartung et al., 2016; Wimmer et al., 2021). Existing experiments focus predominantly on narrative perspective, with considerably less attention to focalization mode and notably lacking exploration of potential interactions between these two dimensions. Moreover, these experiments typically employ relatively short, content-limited narrative texts as stimuli, failing to fully capture the complex cognitive processes inherent in novel reading. Although neuroimaging research has demonstrated that reading narrative texts extensively activates classic language networks in frontal and temporal regions (e.g., prefrontal cortex, inferior frontal gyrus, superior temporal gyrus, middle temporal gyrus) as well as temporoparietal junction and medial prefrontal areas associated with theory of mind processing (Altmann et al., 2014; Lehne et al., 2015; Mar, 2011; Zacks et al., 2018), only a handful of studies (e.g., Hartung et al., 2017) have directly compared neural mechanisms underlying different narrative perspectives. Critically, due to methodological limitations (e.g., the use of only two brief stories), existing studies (e.g., Hartung et al., 2017) have yet to reveal significant differences in neural processing across narrative perspectives.

To address these gaps, the present study employs excerpts from classic novels as experimental stimuli, combining self-paced reading with functional near-infrared spectroscopy (fNIRS) to systematically investigate how narrative perspective and focalization mode influence reading processes. Experiment 1 uses self-paced reading to examine cognitive processing differences across perspectives and focalization modes. Experiment 2 employs fNIRS to reveal associated neural mechanisms. Additionally, we examine moderating effects of emotional valence and individual differences in social cognition, providing comprehensive empirical evidence for longstanding narratological debates about perspective distinctions (Booth, 1983; Forstreuter, 1924; Shen, 1996) and the relationship between perspective and focalization (Genette, 1980).

### 1.3. Research Questions and Hypotheses

Based on the theoretical and empirical foundations outlined above, this study tests two primary hypotheses:

**Hypothesis** **1.** *Following narratological theories positing essential differences between narrative perspectives (Forstreuter, 1924; Shen, 1996), first-person narration should demonstrate higher cognitive salience than third-person narration. This enhanced salience should manifest as (a) reduced reading times in behavioral measures, particularly under cognitively demanding conditions; and (b) stronger activation in attention-related brain regions, specifically posterior parietal areas associated with attentional control and allocation. These effects should be particularly pronounced under internal focalization or negative emotional valence conditions, which emphasize characters’ subjective experiences and emotional states.*

**Hypothesis** **2.** *Emotional valence should moderate perspective effects on reading processes (Burton et al., 2004; Child et al., 2020; Mouw et al., 2019). Specifically, negative emotional contexts should amplify processing differences between perspectives, with third-person narration requiring longer reading times and potentially evoking compensatory neural activation compared to first-person narration. Furthermore, these valence-moderated effects should be more pronounced in individuals with higher social cognitive abilities, who may be more sensitive to perspective-based variations in narrative structure.*

## 2. Experiment 1: Self-Paced Reading Study

Experiment 1 employed self-paced reading to investigate how narrative perspective and focalization mode influence online text processing, with additional consideration of individual differences in social cognition. If first-person narration possesses inherently higher cognitive salience, processing times should be consistently shorter than for first-person narration compared to third person narration, regardless of focalization mode or emotional valence. Alternatively, if perspective salience is context-dependent, reading time differences should emerge only under specific focalization or valence conditions.

### 2.1. Method

#### 2.1.1. Participants

Sample size determination followed a priori power analysis using G*Power (version 3.1.9.7) for a 2 × 3 mixed-design ANOVA testing within-between interactions. Following Cohen’s (1988) conventions, we specified medium effect size (f = 0.25), α = 0.05, and power = 0.80, yielding a minimum requirement of 42 participants. To ensure adequate power accounting for potential exclusions, we recruited 115 native Chinese-speaking students. Following predetermined exclusion criteria (incomplete data or accuracy < 75%), the final sample comprised 103 participants (88 females, 15 males; Mage = 18.7 years, SD = 1.05). All participants were native Chinese speakers with normal or corrected-to-normal vision, and all of them provided written informed consent and received monetary compensation. This study complied with the Declaration of Helsinki and was approved by the Ethics Committee of Nanjing Normal University.

#### 2.1.2. Experimental Materials and Design

The study implemented a 2 (narrative perspective: first-person, third-person) × 3 (text type: internal focalization, external focalization, negative valence) mixed factorial design. Narrative perspective served as a within-item variable, while text type constituted a between-item variable. The between-item design for text type was necessitated by the use of authentic classic literary texts, which have fixed narrative structures that cannot be systematically matched across the three focalization/valence conditions while preserving their literary integrity. Materials comprised 72 passages excerpted from five nineteenth-century novels in Chinese translation: *Pride and Prejudice*, *Emma*, *Wuthering Heights*, *Jane Eyre*, and *A Tale of Two Cities* (see example passages and their English translations in Table 1). The corpus included 36 first-person and 36 third-person passages, with 12 passages per text type condition. Passage length was standardized at 120–230 Chinese characters to ensure comparable reading demands.

To control for potential confounds from genre and linguistic variations, we created two equivalent material versions. Version 1 utilized original translations; Version 2 modified only personal pronouns while preserving all other content (e.g., converting third-person “she” to first-person “I” or vice versa). A Latin square design balanced perspective presentation across participants, ensuring each participant encountered only one perspective version per passage.

Prior to the main experiment, an independent sample (N = 108; 36 per group) evaluated materials on three dimensions: text acceptability, focalization mode, and emotional valence. Acceptability ratings (1–5 scale; see Table 2) revealed no significant main effects or interactions (all *p*s > 0.20), confirming that perspective manipulations preserved narrative coherence. Focalization mode assessments validated our categorization: internal focalization texts were perceived as narrated from character perspectives (*p* < 0.01), while external focalization texts were perceived as narrated from observer perspectives (*p* < 0.01). For negative valence texts, perspective attributions showed no significant preference (*p* > 0.30).

In emotional valence assessment (see Table 3; 1–5 scale, higher scores indicating stronger negative emotion), negative valence scores were significantly higher than internal focalization texts (*t*(35) = 3.4, *p* < 0.01) and also significantly higher than external focalization texts (*t*(35) = 5.7, *p* < 0.01).

#### 2.1.3. Procedure and Individual Difference Assessment

Participants completed the self-paced reading task individually in a quiet laboratory setting. Each trial began with a fixation cross (500 ms), followed by text presentation divided into eight regions of interest (ROIs). Participants advanced through ROIs via spacebar presses, with each press revealing the next region while masking the previous one. Reading times for each ROI were recorded as the primary dependent variable. Following each passage, participants answered a comprehension question to ensure attentive reading.

To examine individual differences in social cognition, participants completed the Autism Spectrum Quotient (AQ; Baron-Cohen et al., 2001), a 50-item instrument assessing autistic traits across five dimensions: social skills, attention switching, attention to detail, communication, and imagination. Higher scores indicate greater autistic tendencies (range: 0–50). Following established cutoffs (Baron-Cohen et al., 2001; Ruzich et al., 2015) and considering our sample characteristics, participants scoring above 25 were classified as low social cognitive ability (n = 29, M = 27.97, SD = 2.75), while those scoring below 19 were classified as high social cognitive ability (n = 31, M = 15.03, SD = 2.35). Internal consistency was satisfactory (Cronbach’s α = 0.76).

#### 2.1.4. Data Analysis

Reading times underwent preprocessing to remove incorrect responses, times < 200 ms, and outliers exceeding ±2.5 SDs from the mean. Linear mixed-effects models were constructed using the lme4 package in R, with narrative perspective and text type as fixed effects (dummy coded) and maximal random effects structure, including by-subject random intercepts and slopes for perspective and by-item random intercepts. Model fitting employed stepwise simplification when convergence issues arose. The final model specification was: lmer(RT ~ perspective × text_type + (1 + perspective | subject) + (1 | item)). Given the between-item design for text type, the primary analyses focused on examining narrative perspective effects within each text type condition, rather than directly comparing reading times across the three text type conditions.

### 2.2. Results

#### 2.2.1. Overall Analysis

**Figure 1:**
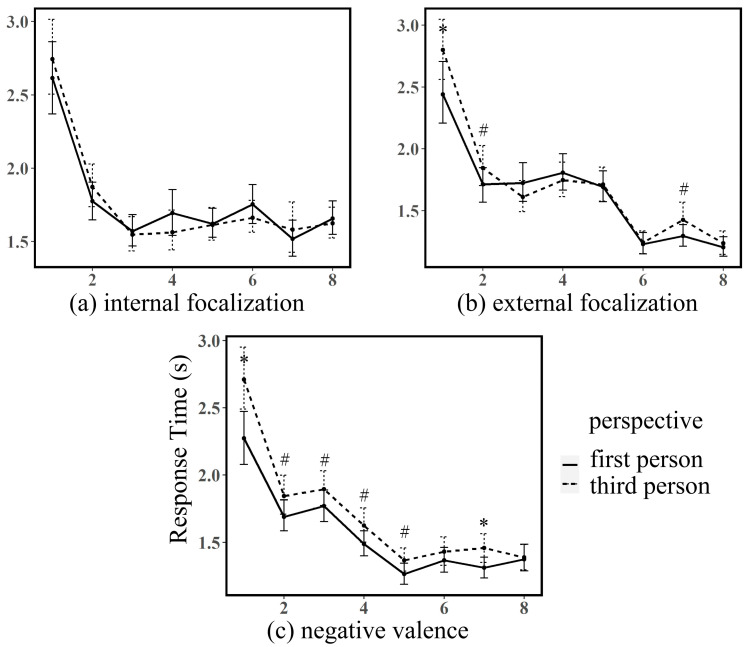
Mean reading times and standard deviations for first-person and third-person texts under different experimental conditions. The horizontal axis represents regions of interest (ROI), and the vertical axis represents participants’ average reaction time (in seconds). * indicates significant differences (*p* < 0.05), ^#^ indicates marginally significant differences (0.05 < *p* < 0.1).

Figure 1 presents mean reading times across ROIs for each experimental condition. Under negative valence conditions, third-person narration consistently produced longer reading times than first-person narration across all regions. This difference reached statistical significance in ROI 1 (*β* = −0.37, *SE* = 0.13, *t* = −2.91, *p* = 0.004) and ROI 7 (*β* = −0.16, *SE* = 0.07, *t* = −2.10, *p* = 0.037), with marginal significance in ROIs 2–5 (all *p*s < 0.10). Internal focalization conditions showed no significant perspective effects across any ROI (all *p*s > 0.10). External focalization conditions yielded an inconsistent pattern: ROI 1 showed significantly longer third-person reading times (*β* = −0.29, *SE* = 0.14, *t* = −2.11, *p* = 0.04), while ROI 3 showed a non-significant reversal. Detailed results are provided in Table 4.

#### 2.2.2. Individual Differences in Social Cognition

**Figure 2:**
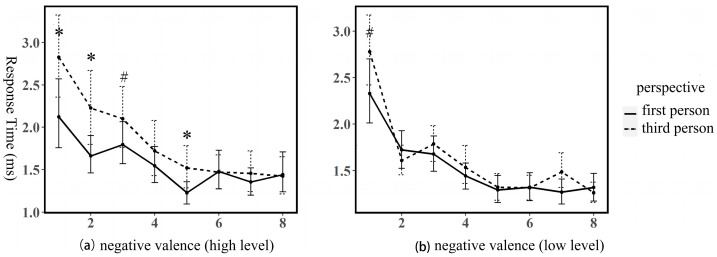
Mean reading times and standard deviations for first-person and third-person texts under negative valence conditions. (**a**) shows results for the high social cognitive ability group, and (**b**) shows results for the low social cognitive ability group. * indicates significant differences (*p* < 0.05), ^#^ indicates marginally significant differences (0.05 < *p* < 0.1).

To explore social cognitive ability as a moderator, we analyzed perspective effects separately for high and low AQ groups under negative valence conditions (Figure 2). The high social cognitive ability group (low AQ) showed pronounced perspective effects, with significantly longer third-person reading times in ROI 1 (*β* = 0.65, *SE* = 0.29, *t* = 2.27, *p* = 0.023), ROI 2 (*β* = 0.55, *SE* = 0.17, *t* = 3.23, *p* = 0.001), and ROI 5 (*β* = 0.33, *SE* = 0.17, *t* = 1.98, *p* = 0.048), and marginal significance in ROI 3 (*β* = 0.28, *SE* = 0.17, *t* = 1.67, *p* = 0.09). The low social cognitive ability group (high AQ) showed only marginal effects in ROI 1 (*β* = −0.31, *SE* = 0.18, *t* = −1.65, *p* = 0.09), with all other comparisons non-significant (*p*s > 0.10). These results indicate that perspective effects were primarily driven by individuals with higher social cognitive abilities.

### 2.3. Discussion

Experiment 1 revealed that narrative perspective significantly influences online text processing, with effects moderated by emotional valence and individual differences. Under negative emotional conditions, third-person narration consistently required longer processing times than first-person narration, particularly among readers with higher social cognitive abilities. These findings suggest that different perspectives engage distinct cognitive processing strategies, with first-person narration demonstrating processing advantages under emotionally charged conditions. The selective emergence of perspective effects under negative valence aligns with theoretical predictions that emotional intensity amplifies structural processing differences. The moderating role of social cognitive ability suggests that sensitivity to perspective manipulations varies with individual differences in social-cognitive processing capacity.

## 3. Experiment 2: Functional Near-Infrared Spectroscopy Study

Experiment 2 employed functional near-infrared spectroscopy (fNIRS) to elucidate the neural mechanisms underlying narrative perspective processing. We addressed the following questions: Do different narrative perspectives and focalization modes elicit distinct patterns of brain activation during novel reading? If so, which brain regions show differential activation, and how does activation intensity vary? Based on theoretical predictions and behavioral findings, we hypothesized that first-person narration would more strongly activate prefrontal and parietal regions associated with attention control (Amodio & Frith, 2006; Rushworth et al., 2001; Saxe & Kanwisher, 2003), with effects potentially enhanced under internal focalization or negative valence conditions. Additionally, we predicted significant interactions in regions supporting social cognition, particularly the temporoparietal junction.

### 3.1. Method

#### 3.1.1. Experimental Design

Experiment 2 maintained the design and materials from Experiment 1, with modifications to accommodate neuroimaging requirements. Each trial began with a 15-s baseline period to establish resting-state brain activity. Based on pilot testing, stimulus presentation duration was fixed at 15 s per passage to ensure adequate hemodynamic response while maintaining ecological validity. Following each passage, participants responded to a comprehension question via button press to verify attentive reading.

#### 3.1.2. Participants

Forty-one right-handed native Chinese speakers (age range: 19–25 years) with no history of neurological disorders or reading disabilities participated. Four participants were excluded due to comprehension accuracy below 75%, yielding a final sample of 37 participants (24 females, 13 males). All participants provided written informed consent and received monetary compensation. The study protocol received approval from the Ethics Committee of Nanjing Normal University and complied with the Declaration of Helsinki guidelines.

#### 3.1.3. fNIRS Data Acquisition and Preprocessing

**Figure 3:**
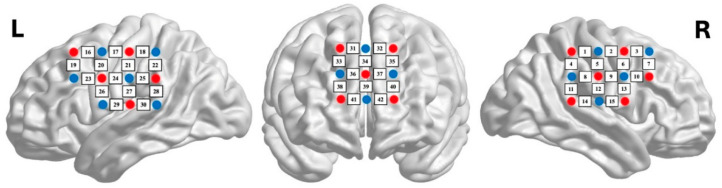
Near-infrared experiment included three probe sets: one placed in the frontal region, and two placed in the left and right hemispheres, respectively (red dots represent emitters, blue dots represent detectors).

Hemodynamic data were collected using a 32-fiber LABNIRS (Shimadzu Corporation, Kyoto, Japan) with a temporal resolution of 99 ms and a sampling frequency of 10.101 Hz. The probe configuration comprised 16 emitters and 15 detectors arranged to create 43 measurement channels covering bilateral temporoparietal and prefrontal regions (Figure 3). Source-detector separation was maintained at 3 cm. Three wavelengths (780 nm, 805 nm, 830 nm) were recorded to calculate oxygenated (HbO) and deoxygenated (HbR) hemoglobin concentrations. Anatomical localization followed the international 10–20 system, with probe positions registered to Montreal Neurological Institute (MNI) coordinates using a 3D digitizer.

fNIRS data preprocessing was completed through the Homer3 toolbox (v1.86). Data from all channels were assessed, and raw voltage signals were converted to optical density data. Wavelet motion correction procedures were applied to correct motion artifacts in optical density data (Molavi & Dumont, 2012). Subsequently, data were filtered using a 0.01–0.1 Hz band-pass filter. General linear models (GLM) were constructed through the MATLAB-based NIRS-KIT toolbox for individual-level statistical analysis to estimate each participant’s HbO *β* coefficients when reading each context (Hou et al., 2021).

Group-level analysis was performed using R software (version 4.2.2). Linear mixed-effects models were constructed through the lmer() function in the lme4 (version 1.1-30) package, with “narrative perspective” and “text type” plus their interaction as fixed effects, establishing a maximal subject-based random effects model.

### 3.2. Results

#### 3.2.1. Behavioral Performance

Participants’ question-answering accuracy exceeded 90%, indicating they could comprehend sentences well and complete experimental tasks.

#### 3.2.2. Neural Activation Patterns

**Figure 4:**
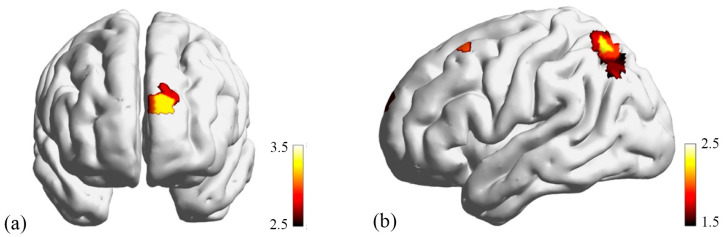
(**a**) In the left frontopolar cortex (Ch40 and Ch42), text type showed a significant main effect; (**b**) First-person narration elicited significantly higher brain activation than third-person narration in the left superior parietal lobule (Ch22).

In the left superior parietal lobule (SPL), narrative perspective showed a significant main effect (Ch22: χ^2^ = 5.36, *p* = 0.020), with first-person narration eliciting stronger brain activation than third-person narration [*t*(195) = 2.32, *p* = 0.022] (see Figure 4b); other effects were all non-significant (*p*s > 0.1). In the left frontopolar cortex, the main effect of text type was significant [Ch40: χ^2^ = 10.05, *p* = 0.016; Ch42: χ^2^ = 10.63, *p* = 0.016]. Post hoc comparisons showed that compared to external focalization, internal focalization elicited stronger activation [Ch40: *t*(195) = 2.80, *p* = 0.015; Ch42: *t*(195) = 3.23, *p* = 0.004; see Figure 4a]. Negative valence texts also showed enhanced activation relative to external focalization (Ch40: *t*(195) = −2.68, *p* = 0.021), but no difference existed between internal focalization and negative valence contexts (*p*s > 0.2).

Although an interaction between perspective and focalization mode emerged in the left superior parietal lobule [Ch27: χ^2^ = 7.07, *p* = 0.029], follow-up analyses with correction revealed no significant differences between the two narrative perspectives under internal focalization, external focalization, or negative valence conditions (all *p*s > 0.10).

### 3.3. Discussion

fNIRS results provide neural evidence for distinct processing mechanisms underlying narrative perspective and focalization mode. First-person narration’s enhanced activation in the left superior parietal lobule aligns with its hypothesized higher cognitive salience, suggesting greater attentional resource allocation. The parietal cortex’s established role in attention control and resource distribution (Corbetta & Shulman, 2002; Rushworth et al., 2001) supports the interpretation that first-person narration captures and maintains attention more effectively than third-person narration. However, this enhanced parietal activation may also reflect alternative mechanisms, including increased demands for referential tracking (as “I” requires continuous updating of the reader’s perspective alignment) or heightened self-referential processing (as first-person pronouns may automatically engage self-related neural circuits even in fictional contexts).

The frontopolar cortex activation pattern reveals that internal focalization and negative emotional content engage similar neural substrates associated with higher-order cognition, including social cognition, metacognition, and complex decision-making (Amodio & Frith, 2006; Moscovitch & Winocur, 2002). This convergence suggests that both internal focalization and negative emotional processing demand enhanced metacognitive engagement and deeper psychological processing, potentially explaining their similar effects on reading behavior. It should be noted that fNIRS has limited spatial specificity compared to fMRI, and the frontopolar region encompasses multiple functional subregions. Therefore, while the observed activation pattern is consistent with metacognitive and social-cognitive engagement, the precise functional attribution should be interpreted with appropriate caution.

## 4. General Discussion

This study employed complementary behavioral and neuroimaging methodologies to investigate how narrative perspective, focalization mode, and emotional valence shape novel reading processes. The convergent evidence reveals that these narrative dimensions engage distinct cognitive and neural mechanisms, with important implications for understanding literary comprehension. Below, we integrate findings across experiments and consider their theoretical and practical significance.

### 4.1. Differential Processing of Narrative Perspective and Focalization Mode

Our findings provide empirical validation for longstanding narratological debates regarding perspective distinctions. The behavioral advantage for first-person narration under negative emotional conditions, coupled with enhanced parietal activation regardless of valence, supports theoretical positions arguing for significant differences between narrative perspectives (Forstreuter, 1924; Rimmon-Kenan, 2002; Shen, 1996). These results challenge claims that perspective differences are merely superficial (Booth, 1983), providing evidence for measurable cognitive and neural distinctions.

The enhanced parietal activation for first-person narration aligns with its theorized higher cognitive salience. The parietal lobe’s involvement in attention control and resource allocation (Corbetta & Shulman, 2002) suggests that first-person narration commands greater attentional investment. This finding extends previous ERP evidence showing larger P300 components for first-person processing (Brilmayer et al., 2019; L. Chen et al., 2025) by localizing the neural substrate of this attentional enhancement. The stability of this effect across focalization modes indicates that perspective salience operates independently of focalization, supporting hierarchical models of narrative structure.

Internal focalization’s selective activation of the frontopolar cortex reveals its unique cognitive demands. This region’s association with metacognition, theory of mind, and complex social reasoning (Amodio & Frith, 2006) suggests that internal focalization engages deeper cognitive processing than external focalization. The parallel activation pattern for negative emotional content indicates shared neural resources for processing subjective states and emotional information. This convergence may explain why internal focalization and emotional intensity often co-occur in literary texts—both engage similar neurocognitive systems for processing complex psychological content.

The dissociation between behavioral and neural measures warrants consideration. While behavioral effects emerged primarily under negative valence conditions, neural differences appeared across conditions. This pattern suggests that neural measures may be more sensitive to perspective manipulations, detecting differences that do not necessarily manifest in overt behavior.

### 4.2. The Moderating Roles of Emotional Valence and Individual Differences

Emotional valence emerged as a critical moderator of perspective effects, with negative emotions amplifying processing differences. This finding extends previous work demonstrating enhanced processing for negative content (Burton et al., 2004; Mouw et al., 2019) by showing that emotional arousal specifically enhances sensitivity to structural narrative features. The selective emergence of behavioral perspective effects under negative valence aligns with Child et al.’s (2020) findings and supports theoretical predictions that emotional intensity increases cognitive engagement with narrative structure.

The frontopolar activation for negative content parallels that observed for internal focalization, suggesting shared mechanisms for processing emotional and psychological depth. This neural convergence may reflect the ancient insight (e.g., Aristotle, 2002) regarding the “positive role” of negative emotions in narrative—such content engages deeper cognitive and emotional processing systems, potentially enhancing both comprehension and retention. The evolutionary advantage of heightened processing for negative information may extend to narrative contexts, where negative emotional content signals important information requiring careful processing.

Individual differences in social cognition significantly moderated perspective effects, with higher social cognitive ability (lower AQ scores) associated with greater sensitivity to perspective manipulations. This finding suggests that social-cognitive capacity influences readers’ ability to detect and process narrative structural features. The AQ scale’s focus on social communication and perspective-taking abilities may tap into cognitive resources particularly relevant for processing narrative perspective shifts. This individual difference moderation highlights the importance of considering reader characteristics when investigating literary processing and suggests that narrative effects may vary substantially across populations.

The interaction between individual differences and emotional valence in modulating perspective effects suggests a complex relationship between reader characteristics, text features, and processing outcomes. Readers with higher social cognitive abilities may be particularly attuned to perspective cues under emotionally salient conditions, potentially reflecting enhanced integration of structural and emotional information. This finding has implications for understanding variability in literary response and may inform personalized approaches to literary education.

### 4.3. Theoretical and Practical Implications

Our findings contribute to several theoretical debates in narratology and cognitive literary studies. First, the demonstration of distinct behavioral and neural signatures for different narrative perspectives provides empirical support for theoretical positions emphasizing perspective distinctions (Forstreuter, 1924; Shen, 1996) over those minimizing such differences (Booth, 1983). Second, the independent effects of perspective and focalization mode support hierarchical models of narrative structure, where these dimensions operate at different levels of narrative organization. Third, the moderating role of emotional valence highlights the dynamic interaction between narrative structure and content in shaping reading experiences.

From a neurocognitive perspective, our results extend understanding of the neural networks supporting literary reading. The involvement of attention control regions (parietal cortex) in perspective processing and higher-order social cognitive regions (frontopolar cortex) in focalization and emotional processing reveals how narrative techniques map onto fundamental neurocognitive systems. This mapping suggests that literary devices exploit basic cognitive and neural mechanisms, potentially explaining their effectiveness in engaging readers and shaping comprehension.

Practical implications extend to literary education and therapeutic applications of reading. Understanding how narrative perspective and focalization influence processing can inform pedagogical approaches to teaching literary comprehension. For instance, introducing students to perspective shifts through emotionally engaging texts may enhance their sensitivity to narrative structure. Specifically, educators might select first-person narratives with negative emotional content to maximize student engagement during initial instruction, then gradually introduce third-person perspectives to develop more flexible comprehension strategies. Furthermore, the finding that individuals with higher social cognitive abilities show greater sensitivity to perspective effects suggests that narrative perspective analysis could serve as a valuable tool for developing social-cognitive skills in educational settings. Additionally, the differential engagement of attention and social-cognitive systems by various narrative techniques suggests potential therapeutic applications, where specific narrative forms might be selected to target particular cognitive or emotional processes.

### 4.4. Limitations

Several limitations merit consideration when interpreting our findings. First, narrative perspective was manipulated as a within-item variable and text type (focalization mode and emotional valence) as a between-item variable. This design was necessitated by the use of authentic classic literary texts with fixed narrative structures that cannot be systematically matched across the three conditions while preserving literary authenticity. Consequently, our primary analyses of behavioral results focused on comparing perspective effects within each text type condition rather than directly comparing processing across text types. While this design choice reflected constraints imposed by limited suitable literary materials, future studies employing within-item designs could provide more powerful tests of focalization and valence effects.

Second, although we controlled passage length, the complexity and authenticity of classic literary texts precluded systematic matching of other linguistic variables such as syntactic complexity and lexical frequency across conditions. These uncontrolled variables may have contributed to observed differences. However, the consistency of perspective effects across multiple passages from different novels suggests robust findings that are not attributable to idiosyncratic linguistic features of individual texts. Future research using constructed materials could achieve tighter control over these linguistic parameters while potentially sacrificing ecological validity.

Third, the interdependence of focalization mode and emotional valence manipulations complicates the interpretation of their separate and combined effects. Future research should develop materials allowing orthogonal manipulation of these factors to disentangle their contributions. Fourth, the AQ-based subgrouping approach used to examine individual differences warrants cautious interpretation. Dividing a normative sample into “high” and “low” social cognition groups using fixed cutoffs may artificially inflate between-group differences, as this categorical approach discards variance information and creates groups from a continuous distribution. Future research could employ dimensional approaches (e.g., treating AQ scores as continuous predictors in regression models) to more accurately characterize the relationship between social-cognitive abilities and perspective sensitivity.

## 5. Conclusions

This study provides converging behavioral and neural evidence for distinct processing mechanisms underlying narrative perspective and focalization mode in novel reading. First-person narration demonstrates higher cognitive salience than third-person narration, manifested as reduced reading times under emotional conditions and enhanced parietal activation across conditions. Internal focalization engages frontopolar regions associated with metacognition and social cognition, paralleling activation patterns for emotionally negative content. These effects are moderated by individual differences in social cognitive ability, with more socially adept readers showing greater sensitivity to narrative manipulations.

Our findings bridge literary theory and cognitive neuroscience, providing empirical validation for theoretical distinctions between narrative perspectives while revealing their neural substrates. The results emphasize that narrative perspective and focalization mode are not merely stylistic choices but fundamental determinants of how readers process and experience literary texts. Understanding these mechanisms advances theoretical knowledge of literary comprehension and offers practical insights for literary education and potential therapeutic applications of narrative reading. As interdisciplinary research continues to illuminate the cognitive and neural bases of literary experience, we gain a deeper appreciation for the sophisticated ways narrative techniques shape human understanding and empathy.

## Figures and Tables

**Table 1:** Exemplar Experimental Materials by Text Type and Focalization Mode (only one narrative perspective shown for each focalization type; the other version was created by replacing pronouns only).

Text Type	Example Passage (Regions of Interest Separated by “/”)
Internal Focalization	达西那封信，她简直快要背得出了。/她把每一句话都反复研究过，她对于这个写信人的感情，/一忽儿热了起来，一忽儿又冷了下去。/记起他那种笔调口吻，她到现在还是说不尽的气愤，/可是只要一想到以前怎样错怪了他，错骂了他，/她的气愤便转到自己身上来了。/他那沮丧的情绪反而引起了她的同情。/他的爱恋引起了她的感激，他的性格引起了她的尊敬。/（节选自《傲慢与偏见》） Mr. Darcy’s letter she was in a fair way of soon knowing by heart. She studied every sentence; and her feelings towards its writer were at times widely different. When she remembered the style of his address, she was still full of indignation; but when she considered how unjustly she had condemned and upbraided him, her anger was turned against herself; and his disappointed feelings became the object of compassion. His attachment excited gratitude, his general character respect. (Adapted from *Pride and Prejudice*)
External Focalization	我才坐不下来呢，我抓起我出外穿戴的衣帽，/赶忙下楼一一我面前放开一条路了啊。/一走到正房，我四下张望，/想找个人打听打听卡瑟琳的消息。/屋子里照满了阳光，房门大开着，/可是眼前就是看不见一个人。/我正在踌躇，不知道该马上奔回去呢，还是回转去找我家小姐，/这时候忽然轻轻一声咳嗽把我的注意力吸引到壁炉边。/（节选自《呼啸山庄》） Instead of sitting down, I snatched my outdoor things, and hastened below, for the way was free. On entering the house, I looked about for some one to give information of Catherine. The place was filled with sunshine, and the door stood wide open; but nobody seemed at hand. As I hesitated whether to go off at once, or return and seek my mistress, a slight cough drew my attention to the hearth. (Adapted from *Wuthering Heights*)
Negative Valence	按说，她的心中本该感到已经释去重负，/可是她怎么也不能忘却这一切。/冬日的午后，天色开始渐渐暗下来了，/甚至在这种时候，那些叫人胆战心惊的囚车，/还在街上辚辚驶过。/她的思绪紧随着它们，在死囚中寻觅他的踪迹。/接着，她更紧地搂住她丈夫那真实的躯体，/抖得更厉害了。/（节选自《双城记》） It was so impossible to forget that many as blameless as her husband and as dear to others as he was to her, every day shared the fate from which he had been clutched, that her heart could not be as lightened of its load as she felt it ought to be. The shadows of the wintry afternoon were beginning to fall, and even now the dreadful carts were rolling through the streets. Her mind pursued them, looking for him among the Condemned; and then she clung closer to his real presence and trembled more. (Adapted from *A Tale of Two Cities*)

**Table 2:** Material Validation Results: Acceptability and Focalization Mode Ratings.

	Acceptability		Focalization Mode	
	First-Person	Third-Person	Story Character	Narrator
Internal Focalization	3.88 ± 0.26	4.02 ± 0.20	67.3%	31.4%
External Focalization	4.06 ± 0.22	4.10 ± 0.19	33.5%	64.4%
Negative Valence	4.04 ± 0.27	4.13 ± 0.23	51.1%	48.5%

**Table 3:** Means and standard deviations of emotional valence ratings for different text materials.

	Emotional Valence	
	M	SD
Internal Focalization	2.97	0.81
External Focalization	2.62	0.76
Negative Valence	4.03	0.79

**Table 4:** Statistical Comparison of Reading Time Differences Between Third-Person and First-Person Narration.

ROIs	Negative Valence				Internal Focalization				External Focalization			
	*β*	*SE*	*t*	*p*	*β*	*SE*	*t*	*p*	*β*	*SE*	*t*	*p*
ROI 1	−0.37	0.13	−2.91	0.004 **	−0.019	0.13	−1.48	0.14	−0.29	0.14	−2.11	0.04 *
ROI 2	−0.14	0.08	−1.92	0.06 ^#^	−0.11	0.08	−1.39	0.16	−0.15	0.08	−1.90	0.06 ^#^
ROI 3	−0.12	0.07	−1.70	0.09 ^#^	0.01	0.07	0.16	0.88	0.06	0.08	0.78	0.43
ROI 4	−0.14	0.08	−1.76	0.079 ^#^	0.13	0.08	1.63	0.104	0.01	0.08	0.16	0.87
ROI 5	−0.11	0.058	−1.83	0.067 ^#^	−0.01	0.06	−0.19	0.85	−0.02	0.06	−0.26	0.78
ROI 6	−0.07	0.06	−1.11	0.25	0.07	0.06	1.29	0.20	−0.03	0.06	−0.57	0.57
ROI 7	−0.16	0.07	−2.10	0.037 *	−0.1	0.07	−1.46	0.14	−0.14	0.07	−1.83	0.07 ^#^
ROI 8	−0.02	0.05	−0.44	0.66	0.03	0.05	0.54	0.59	−0.06	0.06	−1.06	0.29

Note: ** *p* < 0.01, * *p* < 0.05, ^#^ 0.05 < *p* < 0.10.

## Data Availability

The Experimental materials, data files, and questionnaires can be found at the Open Science Framework: https://osf.io/w3bma/overview?view_only=c03801a789f04301bd94ea5209d8b1d7 (accessed on 9 November 2025).
